# Association of caffeine consumption with all‐cause and cause‐specific mortality in adult Americans with hypertension

**DOI:** 10.1002/fsn3.4079

**Published:** 2024-03-08

**Authors:** Kun Wang, Ziao Li, Jinshen He

**Affiliations:** ^1^ Department of Orthopedic Surgery Third Xiangya Hospital of Central South University Changsha Hunan China; ^2^ Xiangya Scool of Medicine Central South Univeristy Changsha Hunan China

**Keywords:** caffeine, hypertension, mortality, NDI, NHANES

## Abstract

Coffee is an important beverage that is widely consumed, of which caffeine is the main active ingredient. However, the long‐term relationship between caffeine consumption and mortality in hypertensive patients has rarely been studied. This study analyzed a cohort of 12,093 US adults from the National Health and Nutrition Examination Survey from 1999 to 2018. Caffeine consumption was divided into five groups: no intake, >0 to ≤100, >100 to ≤300, >300 to ≤400 and >400 mg/day. Using multivariable‐adjusted Cox proportional hazards models, this study performed a 20‐year follow‐up analysis (1999–2018). In the fully adjusted model, all caffeine consumers had lower all‐cause mortality compared with no intake, especially in the >300 to ≤400 mg/day group (hazard ratio (HR) 0.71, 95% confidence interval (CI) 0.60–0.84). The result of restricted cubic spline also showed a nonlinear association between caffeine consumption and all‐cause mortality. For cardiovascular disease, mortality decreased only at >400 mg/day (HR 0.63, 95% CI 0.47–0.85). For cancer, diabetes, and kidney disease, only >300 to ≤400 mg/day was significantly associated with decreased mortality: (HR 0.60, 95% CI 0.42–0.67), (HR 0.22, 95% CI 0.07–0.75), and (HR 0.32, 95% CI 0.10–0.96), respectively. Lower all‐cause mortality was observed in non‐Hispanic White, African American, population aged 40 or above, and people with a body mass index <25 kg/m^2^. Our findings indicate a nonlinear association between average caffeine consumption and all‐cause mortality, suggesting that hypertensive patients may benefit from moderate caffeine intake.

## Graphical abstract

The long‐term relationship between caffeine consumption and mortality in hypertensive patients has rarely been studied. Our findings indicate a nonlinear association between average caffeine consumption and all‐cause mortality, suggesting that hypertensive patients may benefit from moderate caffeine intake.

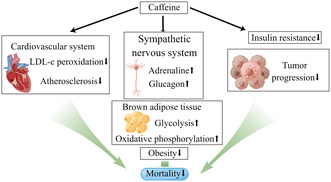

## 1 INTRODUCTION

Hypertension, characterized by abnormally high blood pressure, plays a significant role in the development of cardiovascular disease (CVD) (Lim et al., 2012; Sethi et al., 2022; Tian et al., 2023), diabetes (Sethi et al., 2022), and kidney disease (Teles et al., 2023). Dietary factors are crucial for the prevention and management of hypertension, typically involving recommendations for low sodium and reduced alcohol and fat intake, among others (Ozemek et al., 2018). However, the effects of caffeine‐rich beverages such as coffee on hypertension have remained controversial.

Caffeine (1,3,7‐trimethylxanthine) is the most widely consumed drug worldwide, and its main sources are coffee and tea (McCusker et al., 2003). Many studies have indicated that caffeine consumption correlates with a decrease in all‐cause mortality (Feng et al., 2021; Lin et al., 2022), CVD mortality (Lin et al., 2022), and liver disease mortality (La Vecchia, 2005). However, coffee is not usually recommended for patients with hypertension because it increases blood pressure, total cholesterol, low‐density lipoprotein (LDL) cholesterol, and triglycerides (Heckman et al., 2010).

Nevertheless, a recent study showed an association between moderate caffeine consumption and a reduced risk of all‐cause and cardiovascular mortality in hypertensive patients older than 65 years (Chen et al., 2022). This indicates that individuals with high blood pressure may still benefit from caffeine consumption. Considering the increasing prevalence of hypertension among younger individuals, it is crucial to investigate the effects of caffeine intake on specific age groups outside the elderly population.

To further investigate the association between caffeine intake and all‐cause and cause‐specific mortality in adult hypertensive patients of all ages, this study utilized data from the National Health and Nutrition Examination Survey (NHANES) and the National Death Index (NDI). Mathematical analysis was employed to test the hypothesis that moderate caffeine intake leads to decreased all‐cause mortality and cause‐specific mortality in hypertensive individuals.

## 2 MATERIALS AND METHODS

### 2.1 Study population

**FIGURE 1:**
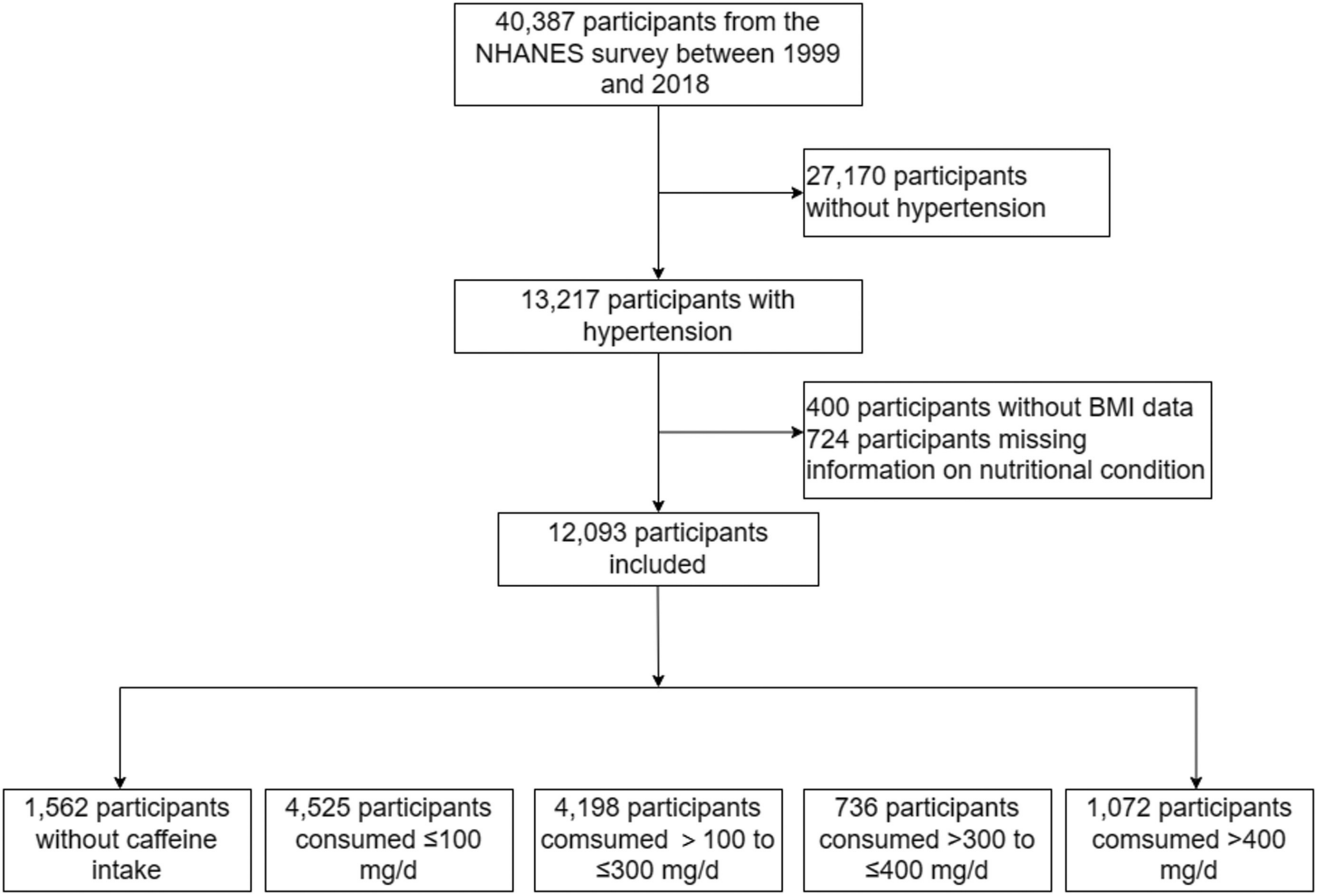
Flow chart of sample selection from the NHANES 1999–2018 (*n* = 12,093). BMI, body mass index; NHANES, National Health and Nutrition Examination Survey.

The data used in this study were obtained from the NHANES conducted between 1999 and 2018, and the participants exclusion process is shown in Figure 1. The NHANES is a comprehensive database with access to the health and nutritional status of adults and children in the USA, while the NDI provides the mortality of adults in the NHANES. The initial sample size was 40,387. After excluding participants without hypertension (*n* = 27,170), body mass index (BMI) data (*n* = 400), and information on nutritional condition (*n* = 724), the number of remaining participants for analysis was 12,093. The follow‐up time was calculated using person‐months from the date of the interview to the date of death or the end of the follow‐up period. This study was approved by the Ethics Review Board of the National Center for Health Statistics, and written consent was obtained from all participants.

### 2.2 Variables

Caffeine intake was the exposure variable of interest in the present study. All NHANES participants were eligible to participate in two 24‐h dietary recall interviews. The first dietary recall interview was conducted in person at the Mobile Examination Center (MEC), and the second interview was conducted by telephone 3–10 days later. To ensure credibility and consistency, the data from the first dietary recall interview were chosen for analysis. Food energy and all nutrients/food components from each food/beverage are calculated using the USDA's Food and Nutrient Database for Dietary Studies. FNDDS is released every two years in conjunction with the WWEIA and NHANES dietary data release. In the 2019–2020 cycle, there are 426 kinds of food containing caffeine, and most of the top 20 foods with the highest caffeine content (from 40 mg/100 g to 5714 mg/100 g) are various types of coffee. The caffeine intake (per day) ranged from 0 mg up to 2448 mg. More detailed information on the caffeine intake data can be found on the website (https://wwwn.cdc.gov/Nchs/Nhanes/2013‐2014/DR1TOT_H.htm). For statistical analysis, the caffeine intake data were divided into five groups based on the level of caffeine intake: no intake, less than 100 mg/day, greater than 100 to ≤300 mg/day, greater than 300 to ≤400 mg/day, and more than 400 mg/day (Feng et al., 2021).

All‐cause and cause‐specific mortality ascertained using the NDI were the outcome variables. Each survey participant eligible for mortality follow‐up was assigned a vital status code (0 = assumed alive; 1 = assumed deceased). The cause of death was determined using the International Classification of Diseases‐10 codes. More details regarding the public‐use linked mortality files can be found in the provided file (https://www.cdc.gov/nchs/data/datalinkage/public‐use‐linked‐mortality‐file‐description.pdf).

Similarly, all covariates were measured using FNDDS. The confounders included in this study can be divided into three categories: basic personal data (such as gender, age, race, BMI, etc.), nutritional status (such as daily intake of fat, protein, energy, etc.), and health status (such as diabetes, asthma, cancer, etc.). Categorical variables included gender, race, income‐to‐poverty ratio, marital status, smoking, alcohol use, drug use for hypertension, diabetes, asthma, congestive heart failure, coronary heart disease, stroke, emphysema, chronic bronchitis, and cancer. The following continuous variables were considered as covariates in this study: age, body mass index, cotinine, plumbum, cadmium, fiber intake, fat intake, energy intake, protein intake, carbohydrate intake, saturated fatty acids intake, and cholesterol intake. These covariates were selected based on previously published studies (Chen et al., 2022; Feng et al., 2021; Liu et al., 2013; Tian et al., 2023). Directed acyclic graph analysis was used to inform the selection and was performed by DAGitty v3.1 (Figure S1).

### 2.3 Statistical analysis

To roughly test the relationship between caffeine intake and mortality, we used the Kaplan–Meier curve and the ‐ln (‐ln(survival)) figure to assess whether the proportionality of the hazard assumption was violated. The results show that there is no violation. To examine the exact association between caffeine intake and mortality, multivariable‐adjusted Cox proportional hazards models were used, and survival curves were generated. To investigate the dose–response relationship with caffeine, restricted cubic spline curves based on logistic regression models were created, with four knots at the 5th, 35th, 65th, and 95th centiles. Subgroup analysis was conducted using stratified multivariable‐adjusted Cox proportional hazards models by following potential factors: gender, race, age, and BMI, which may affect an individual's metabolic rate and sensitivity to caffeine. Using a regression model that treated the median of each category as a continuous variable, a test for trends over increasing caffeine intake categories was carried out. All analyses were performed using EmpowerStats software (version 3.0), and *p* < .05 was considered a significant result. Forest plots were generated using SangerBox software (Shen et al., 2022).

To ensure that the sample size was adequate to draw credible conclusions, we performed an *F*‐test (ANOVA method) using G*power v3.1.9.7. The following parameters were used: a hypothesized effect size of 0.1, an *α* error probability of .05, an association among measurements of 0.5, a power of 0.95, and nonsphericity correction *ε* = 1. The analysis determined that a minimum sample size of 330 was required, whereas our sample size was 12,093, which meant that the sample size was sufficient for drawing reliable conclusions.

## 3 RESULTS

Table 1 outlines the characteristics of the participants with hypertension, categorized according to their daily caffeine intake (no intake, ≤100, >100 to ≤300, >300 to ≤400, >400 mg/day).

**TABLE 1:** The characteristics of participants stratified by daily caffeine intake from the 1999–2018 NHANES.

Characteristics	Caffeine consumption (mg/day)				
	No intake (*N* = 1562)	>0 to ≤100 (*N* = 4525)	>100 to ≤300 (*N* = 4198)	>300 to ≤400 (*N* = 736)	>400 (*N* = 1072)
Age, years	57.7 ± 16.2	61.2 ± 16.0	60.6 ± 15.0	59.7 ± 13.6	56.0 ± 13.7
Body mass index, kg/m^2^	31.4 ± 8.0	30.6 ± 7.2	30.5 ± 6.6	31.1 ± 7.1	31.5 ± 7.2
Male	711 (45.5%)	1913 (42.3%)	1971 (47.0%)	412 (56.0%)	680 (63.4%)
Race					
Mexican American	223 (14.3%)	677 (15.0%)	537 (12.8%)	89 (12.1%)	113 (10.5%)
Hispanic	74 (4.7%)	349 (7.7%)	357 (8.5%)	37 (5.0%)	34 (3.2%)
Non‐Hispanic White	430 (27.5%)	1895 (41.9%)	2255 (53.7%)	495 (67.3%)	799 (74.5%)
African American	752 (48.1%)	1376 (30.4%)	854 (20.3%)	89 (12.1%)	89 (8.3%)
Other races	83 (5.3%)	228 (5.0%)	195 (4.6%)	26 (3.5%)	37 (3.5%)
Income‐to‐poverty ratio^a^					
<1	328 (21.0%)	771 (17.0%)	550 (13.1%)	99 (13.5%)	166 (15.5%)
1–3	585 (37.5%)	1691 (37.4%)	1533 (36.5%)	252 (34.2%)	333 (31.1%)
>3	347 (22.2%)	1247 (27.6%)	1386 (33.0%)	265 (36.0%)	366 (34.1%)
Marital status	752 (48.1%)	2543 (56.2%)	2592 (61.7%)	498 (67.7%)	711 (66.3%)
Cotinine, ng/mL	41.2 ± 108.4	34.9 ± 106.3	52.4 ± 127.2	70.1 ± 140.7	105.3 ± 165.4
Plumbum, μg/dL	2.1 ± 2.2	2.0 ± 1.7	2.0 ± 1.7	2.0 ± 1.6	2.1 ± 1.7
Cadmium, μg/L	0.5 ± 0.5	0.5 ± 0.5	0.5 ± 0.5	0.6 ± 0.7	0.7 ± 0.8
Fiber, g/day	14.4 ± 9.8	15.3 ± 9.7	15.6 ± 9.2	16.3 ± 9.7	16.3 ± 9.9
Fat, g/day	61.7 ± 40.7	67.8 ± 40.2	74.8 ± 41.3	84.3 ± 45.6	94.0 ± 53.6
Energy, kcal/day	1738 ± 923	1818 ± 851	1947 ± 873	2124 ± 917	2373 ± 1163
Protein, g/day	70.7 ± 40.5	71.3 ± 36.2	75.7 ± 37.1	81.1 ± 39.5	87.8 ± 46.6
Carbohydrate, g/day	209.8 ± 114.3	225.6 ± 108.6	235.7 ± 109.7	249.6 ± 115.1	282.9 ± 157.3
Saturated fatty acids, g/day	19.3 ± 14.1	21.9 ± 14.6	24.1 ± 14.5	27.4 ± 16.0	31.0 ± 19.2
Cholesterol, mg/day	258.1 ± 230.4	258.3 ± 209.6	280.5 ± 219.3	312.7 ± 242.4	330.5 ± 261.5
Smoking	673 (43.1%)	1983 (43.8%)	2214 (52.7%)	460 (62.5%)	790 (73.7%)
Alcohol use	861 (55.1%)	2587 (57.2%)	2834 (67.5%)	547 (74.3%)	822 (76.7%)
Drug use for hypertension	1304 (83.5%)	3879 (85.7%)	3581 (85.3%)	630 (85.6%)	846 (78.9%)
Diabetes	414 (26.5%)	989 (21.9%)	983 (23.4%)	139 (18.9%)	224 (20.9%)
Asthma	236 (15.1%)	694 (15.3%)	610 (14.5%)	109 (14.8%)	168 (15.7%)
Congestive heart failure	116 (7.4%)	326 (7.2%)	267 (6.4%)	37 (5.0%)	80 (7.5%)
Coronary heart disease	89 (5.7%)	424 (9.4%)	355 (8.5%)	71 (9.6%)	100 (9.3%)
Stroke	126 (8.1%)	377 (8.3%)	297 (7.1%)	50 (6.8%)	79 (7.4%)
Emphysema	39 (2.5%)	131 (2.9%)	136 (3.2%)	38 (5.2%)	72 (6.7%)
Chronic bronchitis	124 (7.9%)	373 (8.2%)	347 (8.3%)	65 (8.8%)	113 (10.5%)
Cancer	183 (11.7%)	667 (14.7%)	747 (17.8%)	115 (15.6%)	176 (16.4%)

*Note*: Mean ± SD for continuous variables, *p* value was calculated using weighted linear regression model, % for categorical variables, *p* value was calculated using weighted chi‐square test.

^a^ Variables with missing data as another category; the cumulative percentage was not 100%.

**FIGURE 2:**
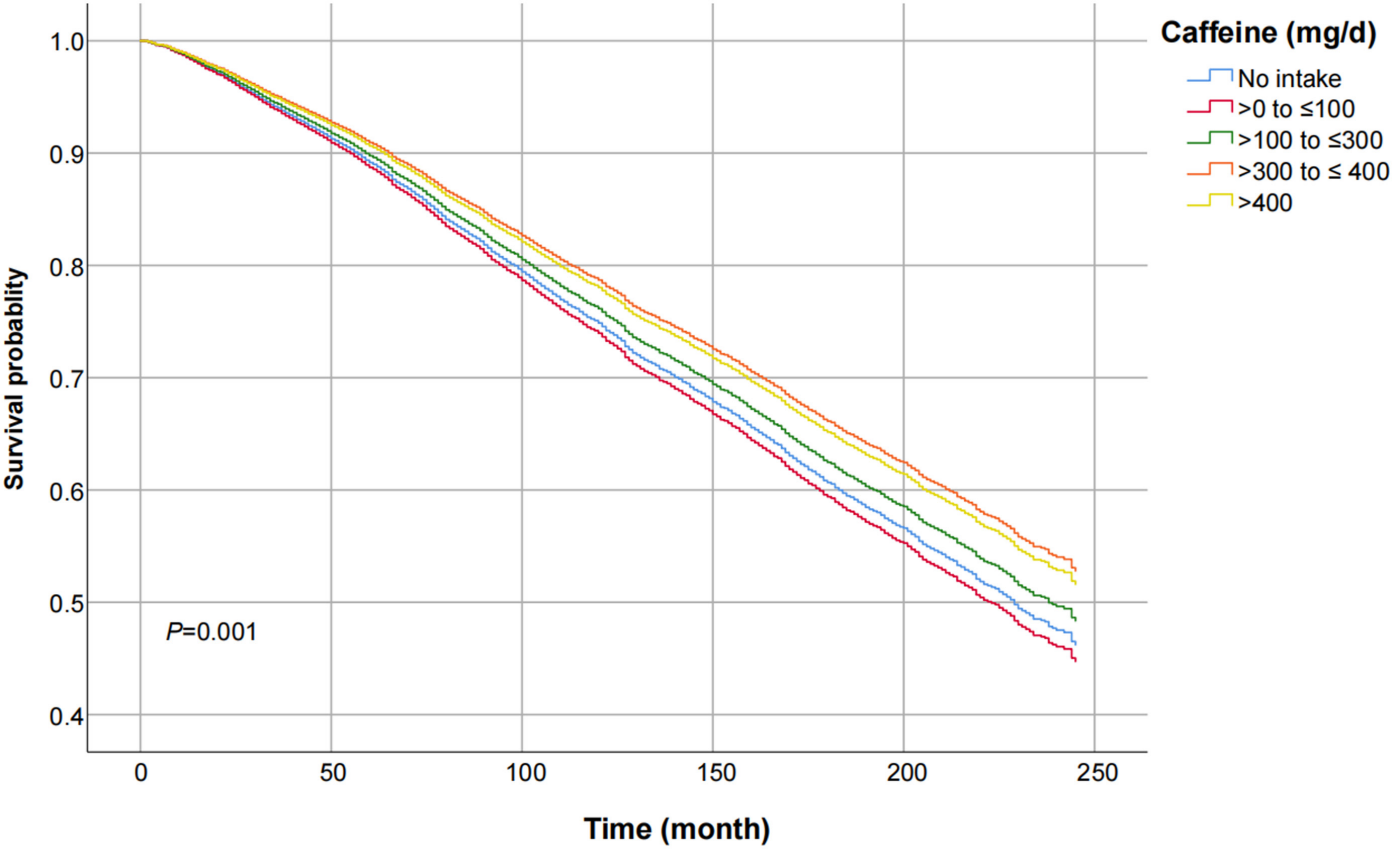
Kaplan–Meier curves for all‐cause mortality by different caffeine consumption groups. CI, confidence ratio; HR, hazard ratio.

**FIGURE 3:**
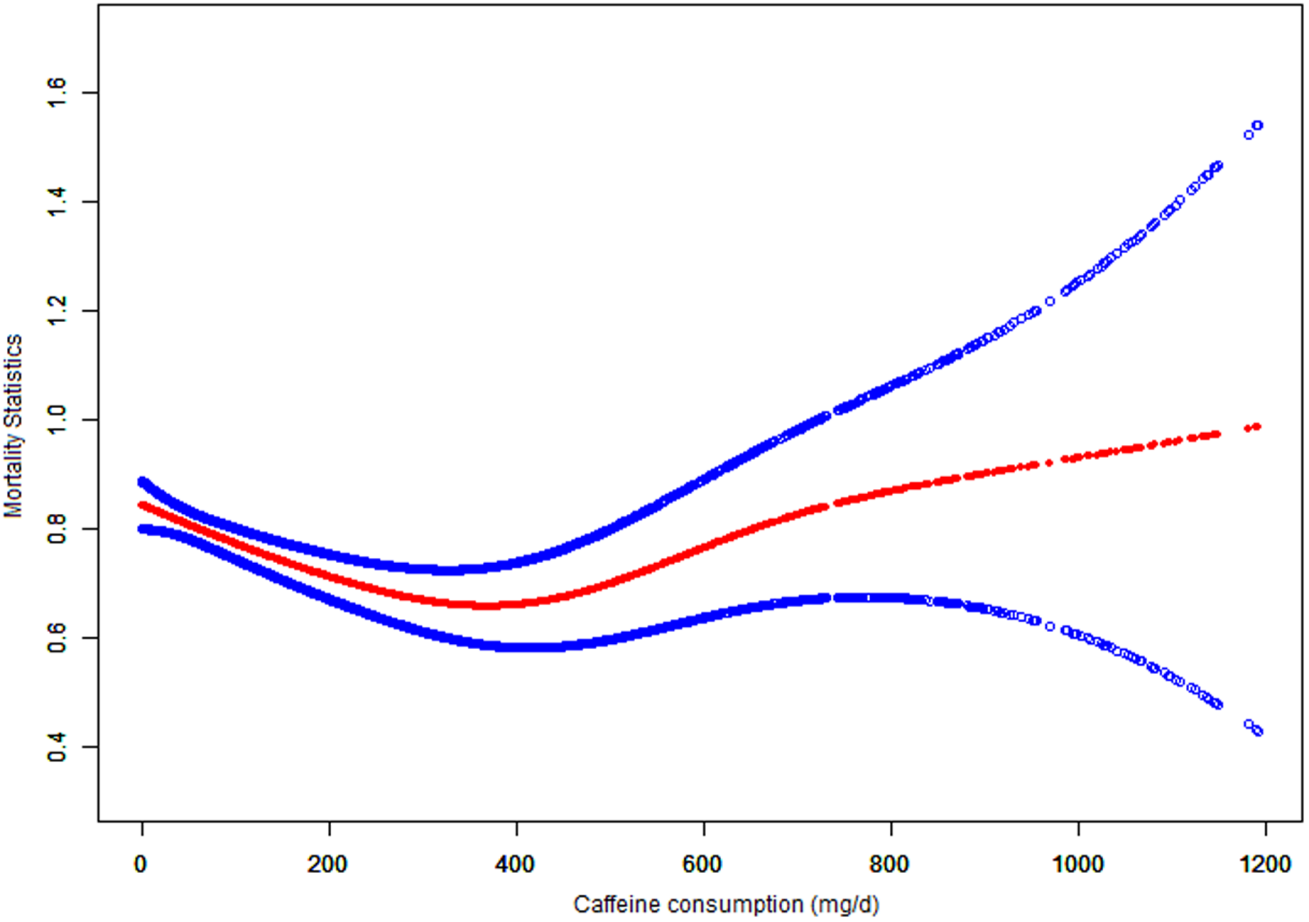
Association between caffeine consumption and all‐cause mortality examined by multivariable Cox regression models based on restricted cubic splines. *p* for nonlinearity is .0148. The middle line represents estimates of hazard ratios. HR was adjusted for gender, age, race, BMI, income‐to‐poverty ratio, marital status, smoking, alcohol use, cotinine, plumbum, cadmium, fiber, fat, energy intake, protein, carbohydrate, saturated fatty acids, cholesterol, drug use for hypertension, diabetes, asthma, congestive heart failure, coronary heart disease, stroke, emphysema, chronic bronchitis, cancer.

Figure 2 shows the crude relationship between caffeine intake and mortality, which demonstrates that a daily intake of 300–400 mg caffeine has the most significant effect on reducing mortality. Table 2 summarizes the results of the multivariable‐adjusted Cox proportional hazards regression analyses of caffeine consumption and all‐cause and cause‐specific mortalities. Initially, without adjusting for confounding variables, caffeine consumption >300 mg/day showed a positive association with the all‐cause mortality. However, after adjusting for BMI, gender, age, and race, the minimally adjusted model indicated a strong association between caffeine intake of ≤400 mg/day and all‐cause mortality. In the fully adjusted model, all caffeine consumers exhibited a significant decrease in all‐cause mortality, particularly among those with a daily intake of 300–400 mg of caffeine (HR = 0.71, 95% CI = 0.60–0.84). The graph in Figure 3 illustrates the nonlinear association between mean caffeine consumption and all‐cause mortality.

**TABLE 2:** Association of caffeine consumption with all‐cause and cause‐specific mortality in hypertensive populations.

Mortality cause	Caffeine consumption (mg/day)					*p* for trend
All‐cause	No intake (*N* = 1562)	>0 to ≤100 (*N* = 4525)	>100 to ≤300 (*N* = 4198)	>300 to ≤400 (*N* = 736)	>400 (*N* = 1072)	
**Deaths**	**520**	**1538**	**1305**	**217**	**323**	
Non‐adjusted	1	1.04 (0.94, 1.15)	0.94 (0.85, 1.04)	0.83 (0.71, 0.97)	0.86 (0.75, 0.98)	0.0001
Minimally adjusted	1	0.83 (0.75, 0.92)	0.79 (0.72, 0.88)	0.72 (0.62, 0.85)	0.99 (0.86, 1.14)	0.1711
Fully adjusted	1	0.86 (0.78, 0.95)	0.81 (0.73, 0.90)	0.71 (0.60, 0.84)	0.82 (0.70, 0.95)	0.0004
**CVD**	**No intake (*N* = 1181)**	**>0 to ≤100 (*N* = 3427)**	**>100 to ≤300 (*N* = 3264)**	**>300 to ≤400 (*N* = 587)**	**>400 (*N* = 824)**	
**Deaths**	**139**	**440**	**371**	**68**	**75**	
Non‐adjusted	1	1.12 (0.93, 1.36)	0.99 (0.82, 1.21)	0.95 (0.71, 1.27)	0.73 (0.55, 0.97)	0.0053
Minimally adjusted	1	0.85 (0.70, 1.03)	0.79 (0.65, 0.97)	0.82 (0.61, 1.10)	0.84 (0.63, 1.13)	0.1512
Fully adjusted	1	0.92 (0.76, 1.12)	0.85 (0.69, 1.04)	0.81 (0.60, 1.10)	0.63 (0.47, 0.85)	0.0019
**Cancers**	**No intake (*N* = 1142)**	**>0 to ≤100 (*N* = 3282)**	**>100 to ≤300 (*N* = 3186)**	**>300 to ≤400 (*N* = 562)**	**>400 (*N* = 841)**	
**Deaths**	**100**	**295**	**293**	**43**	**92**	
Non‐adjusted	1	1.05 (0.84, 1.32)	1.09 (0.87, 1.36)	0.85 (0.60, 1.22)	1.21 (0.91, 1.61)	0.3897
Minimally adjusted	1	0.82 (0.65, 1.03)	0.86 (0.68, 1.08)	0.68 (0.48, 0.99)	1.28 (0.95, 1.73)	0.1600
Fully adjusted	1	0.81 (0.64, 1.03)	0.85 (0.67, 1.08)	0.60 (0.42, 0.87)	0.96 (0.70, 1.30)	0.6213
**Diabetes mellitus**	**No intake (*N* = 1071)**	**>0 to ≤100 (*N* = 3054)**	**>100 to ≤300 (*N* = 2955)**	**>300 to ≤400 (*N* = 522)**	**>400 (*N* = 767)**	
**Deaths**	**29**	**67**	**62**	**3**	**18**	
Non‐adjusted	1	0.84 (0.54, 1.29)	0.80 (0.52, 1.25)	0.20 (0.06, 0.67)	0.83 (0.46, 1.50)	0.1437
Minimally adjusted	1	0.68 (0.44, 1.06)	0.70 (0.44, 1.10)	0.19 (0.06, 0.63)	1.00 (0.54, 1.85)	0.4447
Fully adjusted	1	0.83 (0.53, 1.31)	0.88 (0.56, 1.40)	0.22 (0.07, 0.75)	0.86 (0.46, 1.62)	0.3155
**Kidney diseases**	**No intake (*N* = 1067)**	**≤100 (*N* = 3038)**	**>100 to ≤300 (*N* = 2927)**	**>300 to ≤400 (*N* = 523)**	**>400 (*N* = 757)**	
**Deaths**	**25**	**51**	**34**	**4**	**8**	
Non‐adjusted	1	0.75 (0.46, 1.20)	0.52 (0.31, 0.87)	0.31 (0.11, 0.90)	0.43 (0.19, 0.96)	0.0023
Minimally adjusted	1	0.63 (0.39, 1.02)	0.48 (0.28, 0.82)	0.33 (0.11, 0.96)	0.66 (0.29, 1.52)	0.0399
Fully adjusted	1	0.72 (0.44, 1.20)	0.60 (0.34, 1.04)	0.32 (0.10, 0.96)	0.50 (0.21, 1.20)	0.0267

*Note*: The data: HR (95%CI).

Unadjusted model adjusted for no covariates.

Minimally adjusted model adjusted for: BMI, gender, age, and race.

Fully adjusted model was adjusted for: gender, age, race, BMI, income‐to‐poverty ratio, marital status, smoking, alcohol use, cotinine, plumbum, cadmium, fiber, fat, energy intake, protein, carbohydrate, saturated fatty acids, cholesterol, drug use for hypertension, diabetes, asthma, congestive heart failure, coronary heart disease, stroke, emphysema, chronic bronchitis, and cancer.

Abbreviations: CI, confidence interval; CVD, cardiovascular disease; HR, hazard ratio.

**FIGURE 4:**
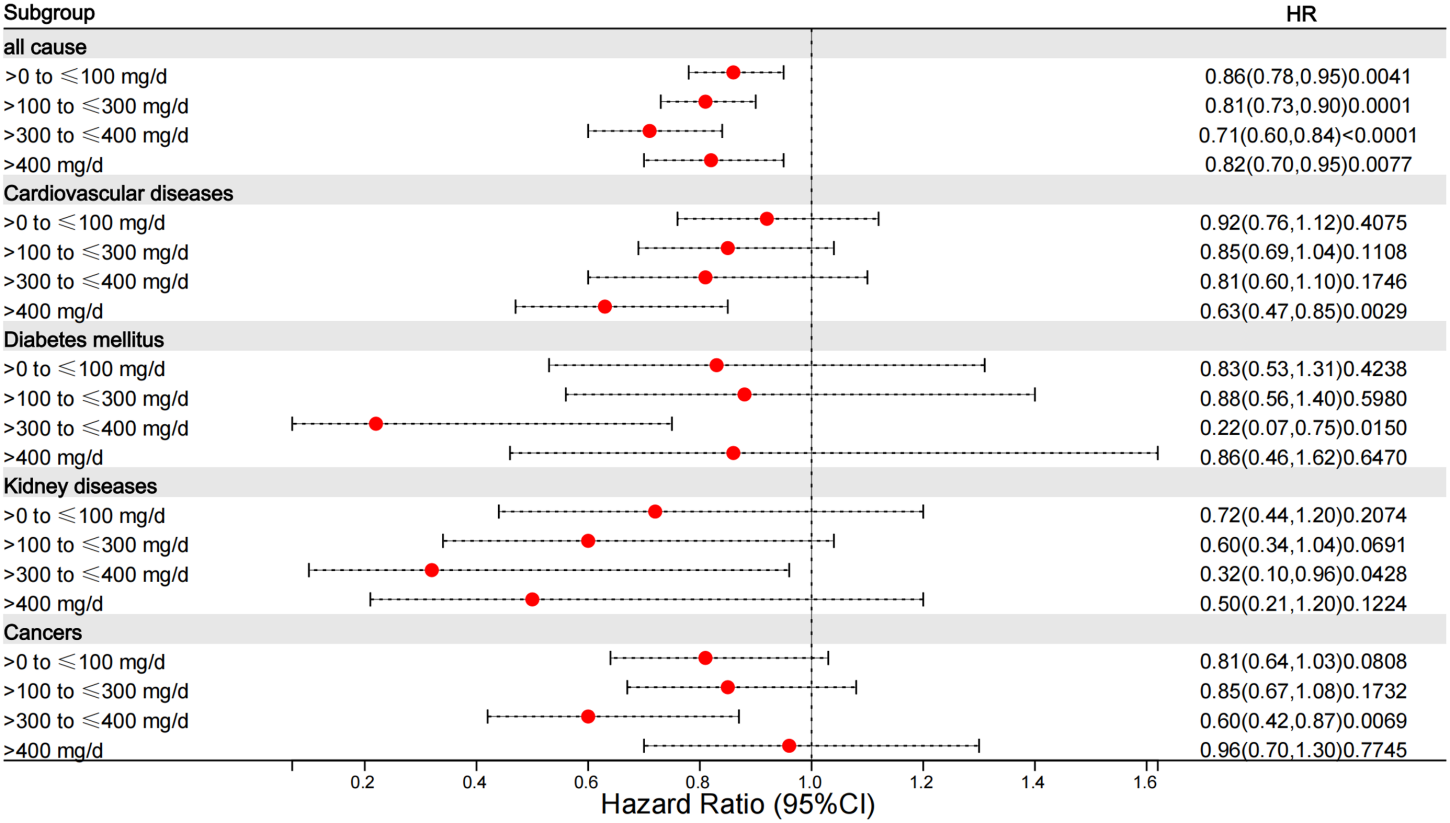
Associations between caffeine consumption and all‐cause and cause‐specific mortality. CI, confidence interval; HR, hazard ratio. HRs were adjusted for gender, age, race, BMI, income‐to‐poverty ratio, marital status, smoking, alcohol use, cotinine, plumbum, cadmium, fiber, fat, energy intake, protein, carbohydrate, saturated fatty acids, cholesterol, drug use for hypertension, diabetes, asthma, congestive heart failure, coronary heart disease, stroke, emphysema, chronic bronchitis, cancer.

The situation regarding cause‐specific mortality was somewhat different. In relation to CVD, mortality only decreased in individuals with caffeine intake exceeding 400 mg/day in the fully adjusted model (HR = 0.63, 95% CI = 0.47–0.85). As for cancer, there was a significant decrease in mortality among participants with a daily consumption of 300–400 mg caffeine in the fully adjusted model (HR = 0.60, 95% CI = 0.42–0.87). In terms of diabetes, mortality declined among those who consumed 300–400 mg/day of caffeine in the fully adjusted model (HR = 0.22, 95% CI = 0.07–0.75). The pattern observed in kidney diseases was similar to that observed in diabetes, with significant associations found in the fully adjusted model (HR = 0.32, 95% CI = 0.10–0.96). It is important to note that the results for diabetes and kidney disease need to be treated with caution, as the number of deaths is very low in the caffeine group 300–400 mg/day, and may not be enough to draw firm conclusions. Figure 4 provides an overview of the association between caffeine intake and all‐cause and cause‐specific mortalities.

The subgroup analyses, which were stratified by gender, race, age, and BMI, are presented in Table 3. Among males, all‐cause mortality decreased only in those consuming 300–400 mg/day of caffeine (HR = 0.80, 95% CI = 0.64–0.99). Conversely, among females, all women who consumed caffeine experienced a decrease in all‐cause mortality, particularly at a daily intake of 300–400 mg (HR = 0.58, 95% CI = 0.45–0.75). Non‐Hispanic whites exhibited a decline in all‐cause mortality among those with a caffeine intake ranging from 100 to 400 mg/day. African Americans, on the other hand, experienced decreased all‐cause mortality among those consuming caffeine of no more than 300 mg/day. Participants aged 40–59 years also experienced a decrease in all‐cause mortality when they consumed caffeine within the range of 100–400 mg/day. Those over 60 years of age had decreased mortality among individuals with an intake of more than 100 mg/day, particularly when consumption exceeded 400 mg/day (HR = 0.66, 95% CI = 0.55–0.78). In terms of BMI, mortality significantly decreased among individuals consuming 100–300 mg/day of caffeine with a BMI of less than 18.5 kg/m^2^ (HR = 0.36, 95% CI = 0.13–0.99). Those with a BMI of 18.5–24.9 kg/m^2^ experienced a decrease in all‐cause mortality among individuals consuming caffeine but no more than 400 mg/day, especially at 300–400 mg/day (HR = 0.55, 95% CI = 0.38–0.79). However, there was no significant association between caffeine consumption, Mexican Americans, Hispanics, individuals aged 18–39 years, individuals with a BMI over 25 kg/m^2^, and a decrease in all‐cause mortality.

**TABLE 3:** Association of caffeine consumption with all‐cause stratified by gender, race, age and BMI.

Subgroup	Caffeine consumption (mg/day)					*p* for trend
	No intake (*N* = 1562)	>0 to ≤100 (*N* = 4525)	>100 to ≤300 (*N* = 4198)	>300 to ≤400 (*N* = 736)	>400 (*N* = 1072)	
Gender						
Male (*N* = 5687)	1	0.88 (0.77, 1.02)	0.87 (0.75, 1.01)	0.80 (0.64, 0.99)	0.89 (0.73, 1.07)	0.1812
Female (*N* = 6406)	1	0.83 (0.71, 0.95)	0.74 (0.64, 0.87)	0.58 (0.45, 0.75)	0.71 (0.55, 0.92)	<0.0001
Race						
Mexican American (*N* = 1639)	1	0.98 (0.73, 1.32)	1.16 (0.85, 1.59)	1.02 (0.63, 1.65)	0.77 (0.46, 1.30)	0.9373
Hispanics (*N* = 851)	1	0.82 (0.45, 1.49)	0.73 (0.39, 1.36)	0.51 (0.19, 1.38)	1.54 (0.56, 4.26)	0.6308
Non‐Hispanic White (*N* = 5874)	1	0.87 (0.74, 1.02)	0.81 (0.69, 0.95)	0.70 (0.56, 0.87)	0.88 (0.72, 1.07)	0.0602
African American (*N* = 3160)	1	0.84 (0.71, 0.99)	0.72 (0.59, 0.87)	0.68 (0.44, 1.04)	0.56 (0.35, 0.88)	0.0001
Age, years						
18–39 (*N* = 1417)	1	0.85 (0.40, 1.80)	1.18 (0.56, 2.47)	0.96 (0.24, 3.80)	1.12 (0.40, 3.13)	0.6678
40–59 (*N* = 3725)	1	0.88 (0.68, 1.15)	0.75 (0.58, 0.99)	0.52 (0.33, 0.80)	0.99 (0.72, 1.35)	0.5246
≥60 (*N* = 6951)	1	0.95 (0.84, 1.06)	0.87 (0.77, 0.98)	0.75 (0.63, 0.90)	0.66 (0.55, 0.78)	<0.0001
BMI, kg/m^2^						
<18.5 (*N* = 98)	1	0.36 (0.13, 0.99)	0.42 (0.10, 1.80)	0.05 (0.00, 1.30)	1.43 (0.23, 9.05)	0.5098
18.5–24.9 (*N* = 2184)	1	0.82 (0.67, 1.00)	0.76 (0.62, 0.95)	0.55 (0.38, 0.79)	0.72 (0.52, 1.00)	0.0043
25–30 (*N* = 4072)	1	0.95 (0.79, 1.14)	0.87 (0.71, 1.05)	0.78 (0.59, 1.05)	0.88 (0.67, 1.15)	0.0801
>30 (*N* = 5739)	1	0.89 (0.76, 1.05)	0.86 (0.72, 1.01)	0.79 (0.62, 1.01)	0.90 (0.72, 1.12)	0.1998

*Note*: The data: HR (95%CI).

Adjusted for: income‐to‐poverty ratio, marital status, smoking, alcohol use, cotinine, plumbum, cadmium, fiber, fat, energy intake, protein, carbohydrate, saturated fatty acids, cholesterol, drug use for hypertension, diabetes, asthma, congestive heart failure, coronary heart disease, stroke, emphysema, chronic bronchitis, and cancer.

Abbreviations: BMI, body mass index; CI, confidence interval; HR, hazard ratio.

## 4 DISCUSSION

In our study, inverse associations were observed between caffeine consumption and all‐cause CVD, cancer, diabetes, and kidney disease mortality in hypertensive patients. Specifically, daily consumption of 300–400 mg was significantly associated with reduced mortality, consistent with previous studies (Ale‐Agha et al., 2018; Chen et al., 2022; Li et al., 2021; Liu et al., 2012). Furthermore, intake of >400 mg/day of caffeine was found to decrease mortality due to CVD. However, for African American patients, a decrease in all‐cause mortality was only observed with a consumption of less than 300 mg/day.

Several studies have suggested an association between coffee or caffeine consumption and mortality, both overall and cause‐specific (Chen et al., 2022; Feng et al., 2021; Geleijnse, 2008; Gokcen & Sanlier, 2019; Torres‐Collado et al., 2021), but few have studied conditions in hypertensive patients. A study based on HARVEST reported an increased risk of cardiovascular events associated with coffee consumption in individuals with hypertension (Palatini et al., 2016). However, this study did not investigate the associations with other cause‐specific mortalities. Therefore, this study is the first to comprehensively examine the association between caffeine intake and mortality in adult hypertensive patients.

Although caffeine is generally believed to increase blood pressure, several studies have found that long‐term caffeine consumption does not have this effect (Heckman et al., 2010; Surma & Oparil, 2021) and may even have the opposite effect (Palatini et al., 2009). This finding may partially explain the reduced all‐cause mortality associated with caffeine consumption in hypertensive patients. Additionally, caffeine has a wide range of effects on the body's circulatory, nervous, and immune systems (Chen et al., 2022), which may contribute to a reduction in all‐cause mortality. However, excessive caffeine intake has also been associated with increased all‐cause mortality. This may be due to the stimulatory effect of caffeine on the nervous system, leading to symptoms such as tachycardia, palpitations, insomnia, restlessness, nervousness, tremors, and headaches (Higdon & Frei, 2006). Caffeine is also a diuretic, and long‐term excessive intake may lead to impaired kidney function. In addition, many people drink coffee with the habit of adding vegetable fat or sugar, which may lead to caffeine intake accompanied by excessive intake of sugars and trans‐fatty acids, thereby increasing the risk of CVDs (Feng et al., 2021; Higdon & Frei, 2006).

Regarding cause‐specific mortality, our study results indicate that a higher caffeine intake (>400 mg/day) can help reduce the risk of CVD. Ngueta proposed that caffeine metabolites, especially 7‐methyluric acid, can lower blood pressure, which is the most significant independent risk factor for CVD (Ngueta, 2020). Moreover, caffeine is a kind of antioxidant (Gokcen & Sanlier, 2019; Yamagata, 2018), which helps reduce inflammation and can significantly inhibit LDL‐c peroxidation and prevent atherosclerosis (Yamagata, 2018), thereby reducing the risk of CVD. As for cancer, our results showed that a daily caffeine intake between 300 and 400 mg was inversely correlated with cancer mortality. This may be explained by the antioxidant effect of caffeine, which prevents oxidative DNA damage (Bøhn et al., 2014). Furthermore, caffeine may activate the DNA repair mechanism, promote the inhibition of DNA methylation, and maintain apoptotic system activity to inhibit tumor progression (Morii et al., 2009). In kidney disease, caffeine can promote blood circulation, which in turn accelerates the elimination of substances such as uric acid and has a good protective effect on the kidney (Li et al., 2021; Liu et al., 2012). In terms of diabetes, other phenolic compounds and substances in coffee, such as fenugreek base or magnesium, may improve insulin sensitivity and glucose resistance, thus improving diabetes (Gokcen & Sanlier, 2019; Tian et al., 2023).

**FIGURE 5:**
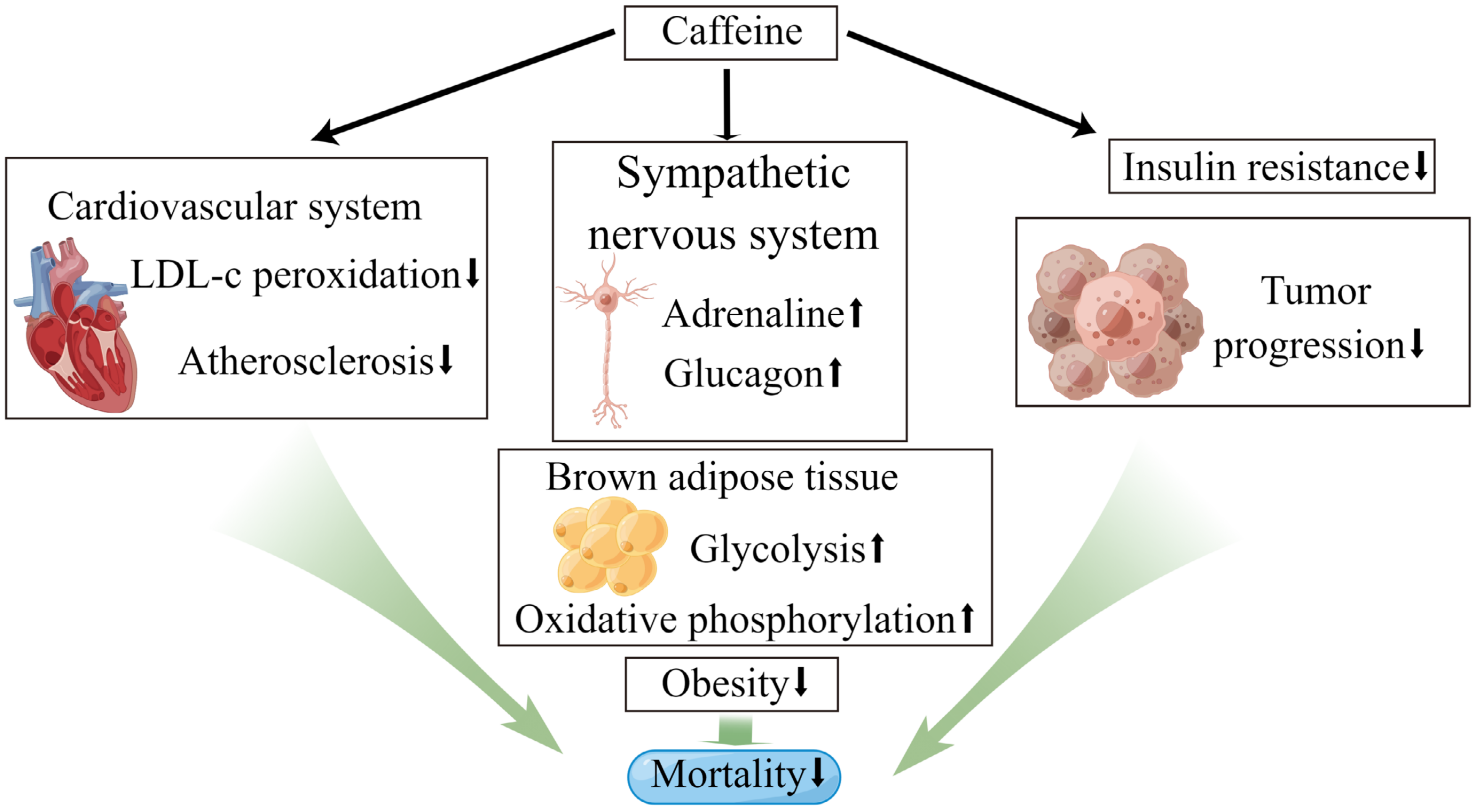
Schematic diagram of possible mechanisms of caffeine on mortality decrease. LDL, low‐density lipoprotein. The figure is drawn by www.figdraw.com.

Finally, in the subgroup analysis, only patients with a BMI <25 kg/m^2^ showed a significant decrease in all‐cause mortality with caffeine intake. Kamimori et al. suggested that thin individuals have a stronger ability to metabolize caffeine, resulting in a smaller amount of caffeine producing more metabolites and easier exertion of its beneficial effects (Kamimori et al., 1987). Additionally, the effects of caffeine differed according to gender, race, and age groups. Women tended to benefit more from caffeine than men, and only non‐Hispanic whites, African Americans, and individuals aged above 40 years showed significant benefits. It is worth noting that our study was based on an American population, and reliable data for the Asian population are not available. Genes (Mielgo‐Ayuso et al., 2019), hormone levels (Temple et al., 2014), and caffeine sensitivity (Jee et al., 2020) might have contributed to these differences. Higher estrogen levels in women may accelerate the absorption of caffeine and its metabolites (Mielgo‐Ayuso et al., 2019). Simultaneously, as age increases, the risk of various diseases gradually increases, which may increase the efficacy of caffeine in reducing mortality. Meanwhile, African Americans and European Americans have different patterns of compensatory sensual‐parasympathetic regulation (Hill & Thayer, 2019), which may affect the effects of caffeine on the regulation of hypertension. Figure 5 provides a brief demonstration of possible mechanisms that could explain the relationship between caffeine intake and mortality.

### 4.1 Strengths and limitations

Based on the NHANES database, this study included a large number of participants from a wide range of races and age groups, which is an advantage of our study. However, this study has several limitations. First, our study only included participants who were 18 years old and above; therefore, the results may not be applicable to younger individuals. Second, certain diseases, such as cancer and kidney disease, in people with a BMI <18.5 kg/m^2^ and African Americans and in groups with more than 300 mg/day caffeine, resulted in a limited number of deaths (less than 100 participants), which may have affected the robustness of the findings. Third, there are sample size differences in gender, race, and BMI stratification, which are due to differences in the American population, and the larger the sample size, the easier it is to detect the difference. As a result, there are overlaps of the confidence intervals across each level in stratification, but significant differences were more likely to occur in the large sample size group. Finally, caffeine consumption was assessed based on a single day of the interview, which may not accurately represent the long‐term intake patterns. Therefore, more precise methods of measuring caffeine intake are necessary to enhance the reliability of the results.

## 5 CONCLUSION

Our findings indicate a nonlinear association between average caffeine consumption and all‐cause mortality, suggesting that moderate intake may be associated with the lowest risk. However, the relationship between caffeine consumption and cause‐specific mortality, as well as subgroup analyses, showed some variations. In most cases, mortality rates decreased among individuals with a daily caffeine intake of 300–400 mg, with the exception of CVDs and all‐cause mortality in African Americans.

## AUTHOR CONTRIBUTIONS

**Jinshen He:** Conceptualization (supporting); funding acquisition (lead); methodology (supporting); project administration (equal); resources (lead); software (lead); supervision (lead); validation (lead). **Kun Wang:** Conceptualization (equal); data curation (lead); formal analysis (supporting); investigation (equal); methodology (equal); project administration (equal); visualization (equal); writing – original draft (equal); writing – review and editing (equal). **Ziao Li:** Conceptualization (equal); data curation (supporting); formal analysis (equal); investigation (equal); methodology (equal); visualization (equal); writing – original draft (equal); writing – review and editing (equal).

## FUNDING INFORMATION

The Wisdom Accumulation and Talent Cultivation Project of the Third Xiangya Hospital of Central South University (YX202209).

## CONFLICT OF INTEREST STATEMENT

The authors declare no conflicts of interest.

## ETHICAL APPROVAL

Approval of this study was obtained from the Ethics Review Board of the National Center for Health Statistics.

## CONSENT FOR PUBLICATION

All the participants provided written informed consent. The experimental protocol was established in accordance with the ethical guidelines of the Declaration of Helsinki.

## Supporting information


Figure S1.

## Data Availability

The data presented in this study are available in the Figshare repository at https://figshare.com/articles/dataset/Caffeine_and_mortality_dataset/22725806.
