# Supplementary figures and images for: Association of caffeine consumption with all‐cause and cause‐specific mortality in adult Americans with hypertension
Source: Food Sci Nutr. 2024 Mar 8;12(6):4185–95. doi: 10.1002/fsn3.4079 (PMC11167170; doi:10.1002/fsn3.4079)

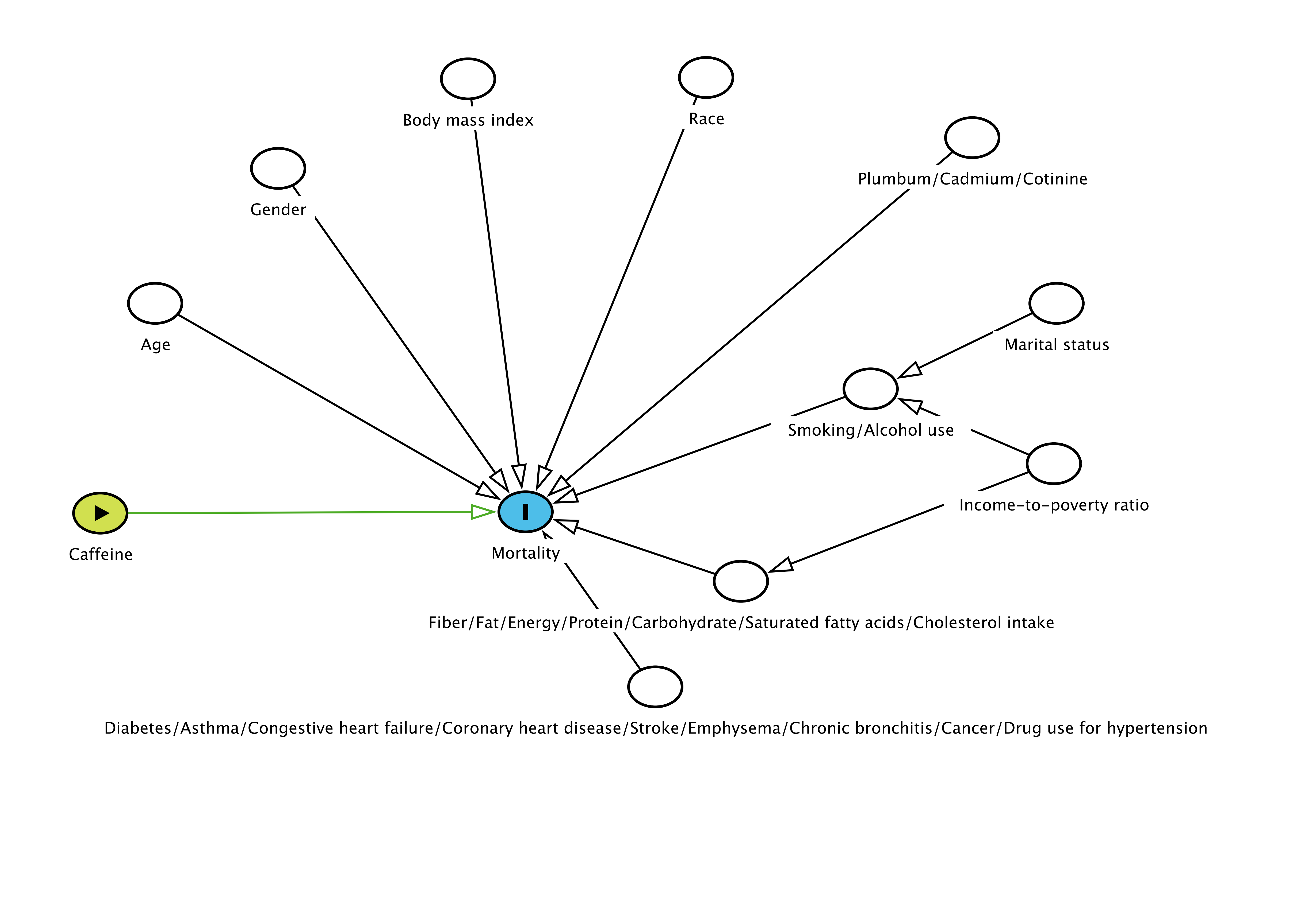

Supplement: Supplementary file 1 — Figure S1. [file FSN3-12-4185-s001.tiff]
